# Ribonucleotide Reductases of *Salmonella* Typhimurium: Transcriptional Regulation and Differential Role in Pathogenesis

**DOI:** 10.1371/journal.pone.0011328

**Published:** 2010-06-25

**Authors:** Anaïs Panosa, Ignasi Roca, Isidre Gibert

**Affiliations:** Institut de Biotecnologia i de Biomedicina and Departament de Genètica i de Microbiologia, Universitat Autònoma de Barcelona, Bellaterra (Cerdanyola del Vallès), Barcelona, Spain; University of Würzburg, Germany

## Abstract

Ribonucleotide reductases (RNRs) are essential enzymes that carry out the *de novo* synthesis of deoxyribonucleotides by reducing ribonucleotides. There are three different classes of RNRs (I, II and III), all having different oxygen dependency and biochemical characteristics. *Salmonella enterica* serovar Typhimurium (*S.* Typhimurium) harbors class Ia, class Ib and class III RNRs in its genome. We have studied the transcriptional regulation of these three RNR classes in *S.* Typhimurium as well as their differential function during infection of macrophage and epithelial cells. Deletion of both NrdR and Fur, two main transcriptional regulators, indicates that Fur specifically represses the class Ib enzyme and that NrdR acts as a global repressor of all three classes. A Fur recognition sequence within the *nrdHIEF* promoter has also been described and confirmed by electrophoretic mobility shift assays (EMSA). In order to elucidate the role of each RNR class during infection, *S.* Typhimurium single and double RNR mutants (as well as Fur and NrdR mutants) were used in infection assays with macrophage and epithelial cell lines. Our results indicate class Ia to be mainly responsible for deoxyribonucleotide production during invasion and proliferation inside macrophages and epithelial cells. Neither class Ib nor class III seem to be essential for growth under these conditions. However, class Ib is able to maintain certain growth in an *nrdAB* mutant during the first hours of macrophage infection. Our results suggest that, during the early stages of macrophage infection, class Ib may contribute to deoxyribonucleotide synthesis by means of both an NrdR and a Fur-dependent derepression of *nrdHIEF* due to hydrogen peroxide production and DNA damage associated with the oxidative burst, thus helping to overcome the host defenses.

## Introduction

Ribonucleotide reductases (RNRs) are essential enzymes that perform the reduction of ribonucleotides (NTPs) to deoxyribonucleotides (dNTPs). This reaction is present in all living organisms and provides the balanced pool of deoxyribonucleotides needed for DNA replication and repair [1].

Three classes of RNRs have been described so far. They differ in the mechanism they use for radical generation, structural differences and oxygen dependence. Class I reductases are strictly aerobic and use an iron center to generate a tyrosyl radical. Class I has been further subdivided into class Ia and class Ib according to structural differences and allosteric regulation (encoded by the *nrdAB* and *nrdHIEF* operons, respectively). The activity of class II reductases (encoded by *nrdJ*) is oxygen-independent due to the use of an adenosylcobalamin cofactor (vitamin B12) to generate the radical. Class III reductases (encoded by the *nrdDG* operon) use S-adenosylmethionine (SAM) and an iron-sulfur cluster to generate a glycyl radical which is extremely sensitive to oxygen and, therefore, class III reductases are strictly anaerobic.

RNRs must be tightly regulated to achieve the dNTPs levels needed to rapidly adapt to any environmental changes. This is accomplished by means of both allosteric and transcriptional regulatory mechanisms. Allosteric mechanisms involve the union of the fully phosphorylated end products of ribonucleotide reduction (dNTPs) to the specificity site, which modulates substrate specificity, and the binding of ATP or dATP to the activity site, which respectively switch on and off the overall activity of the enzyme [2]. Transcriptional regulation has been less studied and little information is available. In *Escherichia coli*, class Ia has been shown to be cell cycle regulated [3], [4] and modulated by several global transcriptional factors such as DnaA, Fis and IciA [5]-[7]. In *E. coli*, class Ib expression has been suggested to be regulated by the transcriptional factor Fur [8], [9], and *nrdDG* expression is anaerobically activated by the global regulator FNR [10]. In the last few years, however, a novel global regulator capable of modulating the expression of all three classes has been extensively described [11]–[13]. Termed NrdR, this protein contains an ATP-cone similar to that found in the activity site of class I and class III RNRs and it has been suggested to modulate RNR expression through the specific sensing of ATP/dATP pools [14].

*Salmonella enterica* serovar Typhimurium (*S.* Typhimurium) is a Gram-negative intracellular human pathogen causing gastroenteritis in humans as well as a systemic infection similar to human typhoid fever in mice. One of the main features of *S. enterica* infection is its capacity to actively invade epithelial cells and proliferate inside macrophages [15].

*S.* Typhimurium contains three different RNRs: class Ia, class Ib and class III, all being biochemically functional with *nrdAB* supporting aerobic growth and *nrdDG* supporting anaerobic growth. *nrdHIEF* however, is poorly expressed and can not complement an *nrdAB* conditional mutant unless a second *nrdHIEF* copy is provided, either as a merodiploid or in a plasmid-based copy [16]. *nrdHIEF* expression, nevertheless, is triggered upon addition of hydroxyurea, growth in minimal media, oxidative stress and iron depletion [8], [9], [16]–[19] suggesting an actual role for this enzyme under certain growth conditions.

This work focuses on the study of the transcriptional regulation exerted by both NrdR and Fur over the three RNR classes present in *S.* Typhimurium. We have studied the effects of an NrdR deletion in RNR expression and provide some insight concerning its role as a main global regulator. We have also analyzed the upregulation of *nrdHIEF* by Fur and detected a Fur recognition sequence within the *nrdHIEF* promoter region. This work also analyzes the role of each RNR during *S.* Typhimurium infection by means of infection assays performed in macrophage and epithelial cell lines.

## Results

### NrdR and Fur negatively regulate *nrd* expression

**Figure 1:**
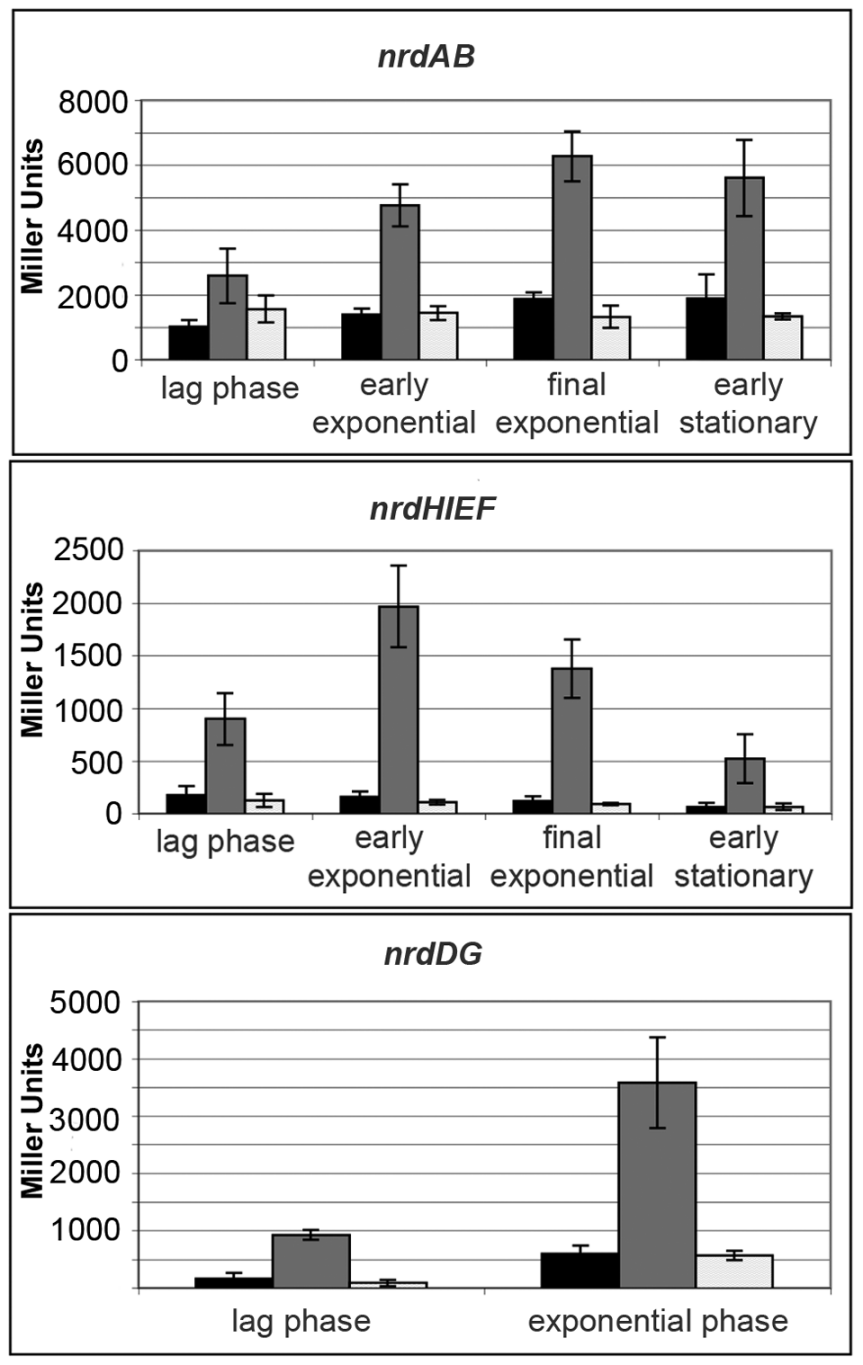
NrdR-dependent expression of RNR. NrdR regulation of *S.* Typhimurium LT2 RNR expression throughout the growth curve (early exponential (OD_550_ = 0.2), final exponential (OD_550_ = 0.8), early stationary phase (OD_550_ = 1.4)). β-galactosidase activity of *nrdAB*, *nrdHIEF*, and *nrdDG* promoter regions fused to *lacZ* expressed in Miller Units (MU). Wild-type strain shown in black, *nrdR* mutant strain in dark grey, and *nrdR* mutant complemented strain in light grey. Results are the mean values of duplicates of at least three independent experiments. Error bars represent the standard deviation of experiments.

In order to assess the transcriptional role of both NrdR and Fur, unmarked *nrdR* and *fur* null mutants of *Salmonella enterica* serovar Typhimurium LT2 were obtained as described in Materials and Methods and then transduced into *Salmonella* strains containing either *nrdAB-lacZ, nrdHIEF-lacZ or nrdDG*-*lacZ* reporter fusions (for *ΔnrdR* mutation) or just *nrdHIEF-lacZ* (for *Δfur* mutation). For the *nrdAB* and *nrdHIEF* operons, expression of the reporter fusions was measured in aerobic conditions throughout the cell cycle. *nrdDG* cultures grew more slowly in anaerobiosis and samples were only taken at lag and exponential phases. The β-galactosidase activity assays (Figure 1) showed an evident upregulation of *nrdAB*, *nrdHIEF* and *nrdDG* expression in the absence of NrdR when compared to the wild-type strains, indicating that NrdR acts as a repressor of all three ribonucleotide reductases in *S.* Typhimurium LT2. *nrdHIEF* expression increased nearly 12-fold compared to the wild-type strain. *nrdAB* and *nrdDG,* however, increased only 3 and 5-fold respectively. Moreover, a growth phase-related differential expression was also observed, being maximal during exponential phase.

**Figure 2:**
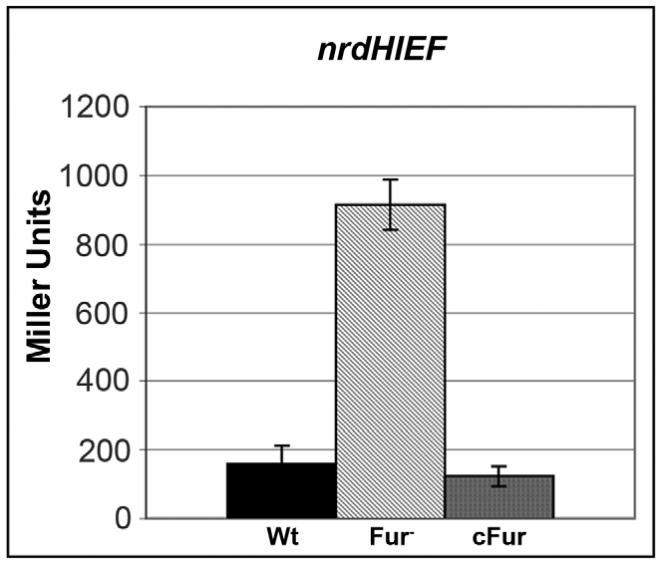
Fur-dependent expression of *nrdHIEF.* Effect of *Δfur* mutation on *nrdHIEF* expression. β-galactosidase activity is expressed in Miller Units (MU) for the wild-type strain (Wt), mutant *Δfur* strain (Fur^-^), and complemented mutant strain (cFur). Results are the mean values of duplicates of at least three independent experiments. Error bars represent the standard deviation of experiments.

As seen in Figure 2, Fur also repressed *nrdHIEF* expression, increasing its transcription up to 5-fold compared to the wild-type strain. *nrdAB* expression was also measured to rule out a global effect of the Fur knock-out on overall gene expression (Figure S1).

To further assess if NrdR and Fur were indeed responsible for this upregulation we performed complementation assays. Both *nrdR* and *fur* were cloned into the pBAD_33_ expression vector under the control of the P_BAD_ promoter and their expression was induced upon addition of 0.3% L-arabinose. β-galactosidase levels for *nrdAB*, *nrdHIEF* and *nrdDG* in the NrdR and Fur complemented strains were similar to those of the wild-type strains, thus supporting their role as transcriptional repressors (Figures 1 & 2, respectively).

### Effect of hydroxyurea in *nrd* expression

Since the late 70s it is well-known that the addition of hydroxyurea (HU) induces the transcriptional expression of both class Ia and class Ib RNR, either in *E. coli*, *S.* Typhimurium or other microorganisms [16], [20], [21].

Hydroxyurea scavenges the tyrosyl radical present in the small subunit of class I enzymes, thus inhibiting its catalytic activity, but it is as yet unknown how such a dramatic downshift in enzymatic activity causes this upregulation in *nrd* transcription.

Being a global *nrd* regulator, we wondered if NrdR might be involved in such phenomenon and, therefore, we performed β-galactosidase assays to analyze the transcriptional levels of *nrdAB, nrdHIEF* and *nrdDG* in the presence and absence of 10 mM HU in both a NrdR mutant and a wild-type strain.

**Figure 3:**
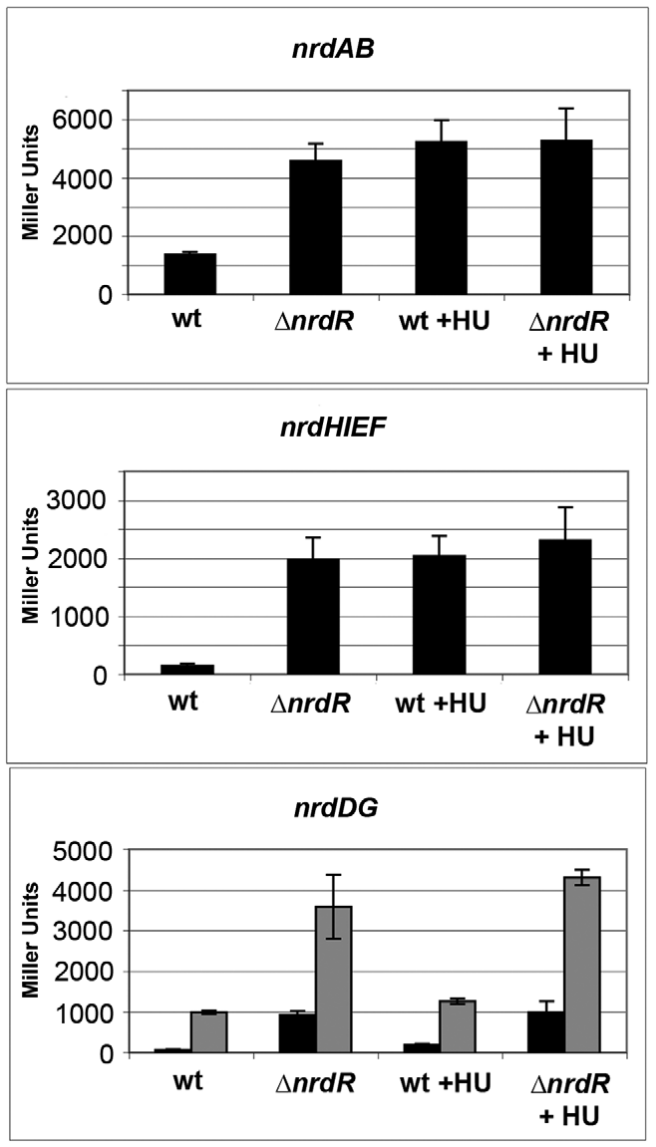
Hydroxyurea-dependent expression of RNR. Effect of hydroxyurea on *nrdAB*, *nrdHIEF* and *nrdDG* expression of wild-type and *ΔnrdR* mutant strains. β-galactosidase activity is expressed in Miller Units (MU). Overnight cultures of each strain were reseeded and grown for 1 hour previous to inoculation with 10 mM hydroxyurea. *nrdDG* fusion strains were grown either in aerobiosis (in black) or anaerobiosis (in grey). Results are the mean values of duplicates of at least three independent experiments. Error bars represent the standard deviation of experiments.

Figure 3 shows that during aerobic growth the induction levels of *nrdAB* and *nrdHIEF* caused by the addition of hydroxyurea were similar to those caused by deletion of NrdR. The addition of hydroxyurea to the *NrdR* mutant did not show any additive increase. Similarly, the β-galactosidase levels of *nrdDG* were very much alike in the NrdR mutant regardless of the presence of hydroxyurea. Wild-type *nrdDG* expression with 10 mM HU showed only a minor increase in both aerobic and anaerobic growth.

### NrdR and Fur recognition sequences

The presence of putative *nrdR* recognition sequences has been studied by phylogenetic profiling in numerous bacterial genomes [22] and it has been established that the NrdR recognition site consists of two tandem NrdR boxes separated by approximately 31–32 bp. In *S.* Typhimurium such boxes can be found within the promoter region of all three *nrd* operons [22], but their actual involvement in *nrd* regulation has not been empirically elucidated.

**Figure 4:**
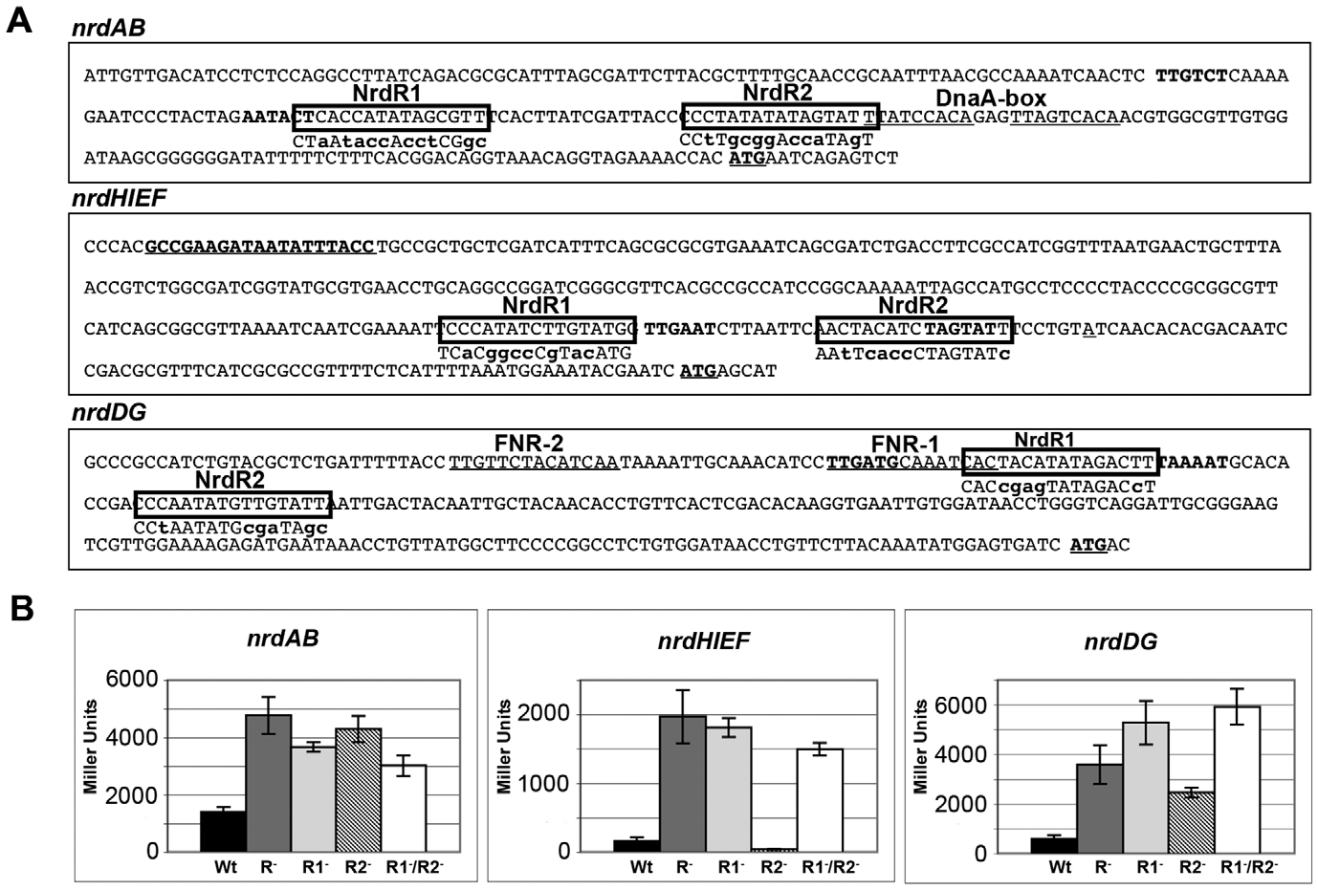
NrdR binding sites. **A.** *S.* Typhimurium LT2 *nrdAB*, *nrdHIEF* and *nrdDG* promoter regions. Black boxes indicate NrdR recognition sites and mutated sequences are showed below in bold lower case. −10 and −35 boxes are showed in bold. Transcriptional start site is indicated in bold and underlined as is the 19 bp Fur box in the *nrdHIEF* promoter region. The DnaA and FNR boxes are underlined for the *nrdAB* and *nrdDG* promoter regions, respectively. **B.** β-galactosidase activities of *nrdAB*, *nrdHIEF*, and *nrdDG* transcriptional fusions with mutagenized NrdR boxes are expressed in Miller Units (MU) for the wild-type strain (Wt), *ΔnrdR* strain (R^−^), NrdR1 box mutant strain (R1^−^), NrdR2 box mutant strain (R2^−^), and NrdR1 and NrdR2 box double mutant strain (R1^−^/R2^−^). Results are the mean values of duplicates of at least three independent experiments. Error bars represent the standard deviation of experiments.

In order to demonstrate the participation of these recognition sequences in NrdR binding and promoter repression, we performed β-galactosidase assays with promoter sequence fusions in which one or both putative NrdR boxes had been significantly altered (single *versus* double mutants, see Figure 4A).

As shown in Figure 4B, modification of either NrdR1, NrdR2 or both, increased *nrdAB* transcription, although only the NrdR2 mutant achieved a transcriptional increase similar to that of the *ΔnrdR* strain.

Modification of the NrdR1 box in the *nrdHIEF* operon resulted in an increase in *nrdHIEF* expression similar to that of the *ΔnrdR* strain. Mutation of the NrdR2 box, however, had no effect on the transcriptional levels of *nrdHIEF* and the double mutant showed transcriptional levels similar to those of the NrdR1 box and the NrdR mutant.

*nrdDG* transcription was greatly induced in both the NrdR1 and NrdR2 mutants, with NrdR2 reaching a similar increase to that of the *ΔnrdR* strain, and NrdR1 even surpassing it. The transcriptional levels of the double mutant paralleled those of the NrdR1 mutant.

**Figure 5:**
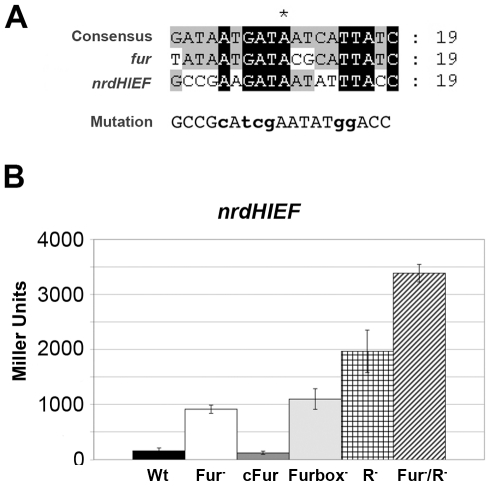
Fur binding sites. **A.** Fur box mutated sequence for the *nrdHIEF* promoter region. Multiple alignment with ClustalW of the *E. coli* Fur box consensus sequence, *S.* Typhimurium LT2 Fur box within the *fur* promoter region and *S.* Typhimurium LT2 Fur box in the *nrdHIEF* promoter region. All sequences were obtained from the NCBI database (http://www.ncbi.nml.nih.gov/). Conserved bases in all three sequences are shown in black, while bases conserved in two out of the three sequences are shown in grey. Base changes in the Fur box sequence are shown in bold lower case. **B.** β-galactosidase activities of the *nrdHIEF*-*lacZ* fusion expressed in Miller Units (MU) for the wild-type strain (Wt), mutant *Δfur* strain (Fur^−^), *fur*-complemented mutant strain (cFur), Fur box mutant strain (Furbox^−^), *ΔnrdR* mutant strain (R^−^) and *ΔfurΔnrdR* double mutant strain (Fur^−^/R^−^). Results are the mean values of duplicates of at least three independent experiments. Error bars represent the standard deviation of experiments.

Vassinova *et al* [9] initially described a putative Fur box within the *E. coli nrdHIEF* promoter region but so far there is no experimental evidence regarding a Fur-mediated regulation of class Ib *nrd* genes. Previous studies in our laboratory indicated an upregulation of *nrdHIEF* under iron depletion and we identified a putative Fur binding box 309 bp upstream from the transcription start site (Figure 4A). To assess its role in the Fur-mediated regulation of *nrdHIEF* we modified the putative Fur box by site-specific mutagenesis (Figure 5A) and monitored the β-galactosidase levels of the *nrdHIEF*-*lacZ* reporter fusion.

As shown in Figure 5B, the transcriptional levels of *nrdHIEF* containing a mutated Fur box were very similar to those obtained with a *Δfur* strain, thus indicating a role for this sequence in *nrdHIEF* regulation.

### Fur protein directly binds the *nrdHIEF* promoter region

To further assess the role of Fur in *nrdHIEF* expression we wanted to demonstrate its direct union to the *nrdHIEF* promoter region. Therefore, we performed electrophoretic mobility shift assays (EMSA) with a 478 bp probe containing the *nrdHIEF* promoter region as well as with another probe containing the same region with a mutated Fur box. As a positive control we used the Fur promoter region since Fur has already been reported to autoregulate its own expression in *E. coli* [23].

**Figure 6:**
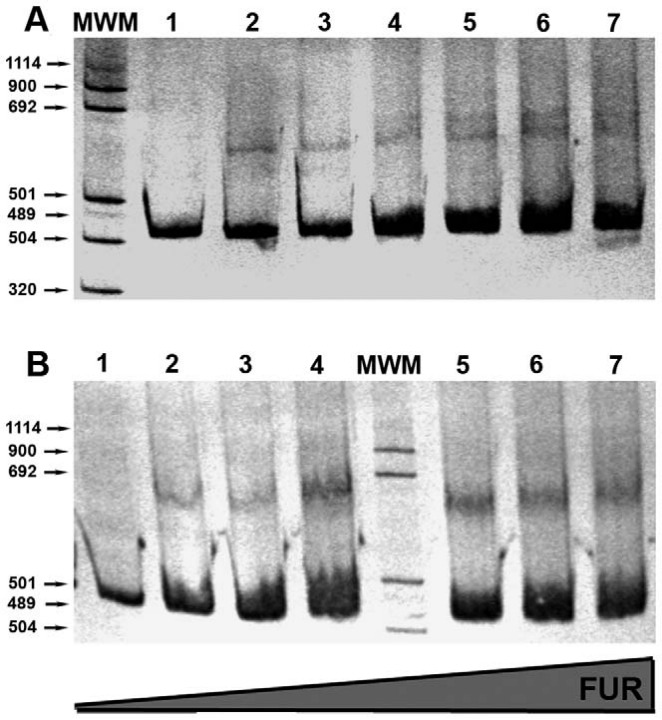
Fur binding to *nrdHIEF*. EMSA of (**A**) *nrdHIEF* and (**B**) *fur* promoter region probes of *S.* Typhimurium LT2, with increasing concentrations of Fur protein: 1–7 lanes: 0, 0.2, 0.4, 0.8, 1, 1.2, and 1.5 µg. MWM: molecular weight marker.

Figure 6 shows the presence of a retarded band when Fur was incubated together with the wild-type promoter, but no mobility shift could be observed when we used a labelled probe containing an altered Fur box (data not shown). A second retarded band seems to appear as protein concentration increases, likely indicating the presence of an additional Fur recognition sequence. Indeed, an additional putative Fur sequence has been identified further upstream from the *nrdHIEF* promoter and its involvement in class Ib transcriptional regulation is in progress.

### Construction of *nrd* mutants

*nrdHIEF* expression levels are usually low and insufficient to maintain cell growth in an *nrdAB* mutant, however, its expression is induced under nutrient starvation, iron depletion and oxidative stress [16], [18], [19], all of which are encountered by *Salmonella* during the course of an infection. This observation, together with the fact that *nrdHIEF* is also regulated by Fur, one of the main modulators of virulence [24]–[26], suggests that *nrdHIEF* may have a leading role in the pathogenesis of *Salmonella.*

We therefore proceeded to evaluate the essentiality of each RNR class during the infectious process, first constructing *nrd* mutants for each RNR class and then combining them to obtain double mutants (Materials and Methods).

Since NrdAB is essential for growth under aerobic conditions, we used two different strategies to construct a class Ia mutant. One *nrdAB* mutant was achieved by inserting an extra copy of *nrdHIEF* elsewhere in the chromosome. The second type of *ΔnrdAB* mutant was constructed during anaerobic growth, where *nrdDG* is used to provide dNTPs.

*ΔnrdEF* (IG138) and *ΔnrdDG* (IG139) mutants were constructed according to the Wanner and Datsenko method (see Materials and Methods).

We also constructed the double mutants *ΔnrdHIEF/ΔnrdDG* (IG140) and *ΔnrdAB/ΔnrdDG*. The latter mutant is not viable, either in aerobiosis or anaerobiosis, unless supplied with an extra *nrdHIEF* copy, but we wanted to check if overexpression of the cognate class Ib enzyme would suffice to maintain growth. Therefore, this double mutant was constructed on both *ΔnrdR* and *Δfur* background strains. We first constructed the *ΔnrdAB/ΔnrdR* (IG143) and *ΔnrdAB/Δfur* (IG144) double mutants grown anaerobically and then transduced the *ΔnrdDG* mutation to originate the triple mutant strains IG145 and IG146, respectively (Table S1). Surprisingly, all *nrdAB* mutants overexpressing *nrdHIEF* proved capable of growing in the presence of oxygen with only a class Ib enzyme, although they displayed much slower growth rates as well as a filamentous morphology (Figure S2).

### Role of RNRs during macrophage infection

*nrd* single and double mutants were used in gentamicin protection assays to infect RAW264.7 mouse macrophage-like cell lines in order to obtain proliferation indexes for each mutant strain.

**Figure 7:**
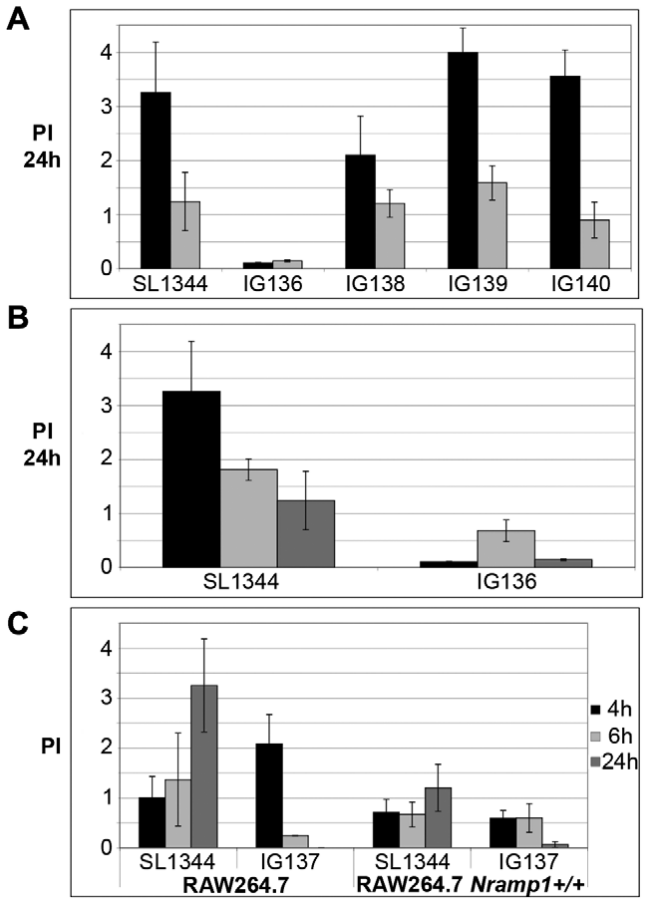
Role of RNR during macrophage infection. **A.** Proliferation indexes (PI) at 24 h p. i. of wild-type, *ΔnrdAB* (IG136), *ΔnrdEF* (IG138), *ΔnrdDG* (IG139), and *ΔnrdEFΔnrdDG* (IG140) mutant strains grown in RAW24.7 (black) or RAW264.7 *Nramp1^+/+^* (grey) mouse macrophage-like cell lines. **B.** Proliferation indexes (PI) at 24 h p.i. of wild-type and *ΔnrdAB* (IG136) strains infecting RAW264.7 (black), RAW264.7 + 65 µM 2,2′-dipyridil (DIP) (light grey) and RAW264.7 *Nramp1^+/+^* (dark grey). **C.** Proliferation indexes (PI) at 4, 6 and 24 h p.i. shown in black, light grey, and dark grey, respectively, of wild-type and IG137 strains infecting RAW264.7 and RAW264.7 *Nramp1^+/+^* mouse macrophage-like cell lines. Results are the mean values of at least three independent experiments. Error bars represent the standard deviation of the mean. P values were determined by Student's *t* test for proliferation indexes. ** indicates significant differences, P<0.05.

As shown in Figure 7A, neither *nrdEF* (PI = 2.1) nor *nrdDG* (PI = 3.99) seemed to be involved in dNTP synthesis during pathogenesis. The *nrdAB* mutant bearing two copies of *nrdHIEF*, however, could not proliferate at 24 h post-infection (PI = 0.105).

The RAW264.7 cell line is defective in the Nramp1 protein, which mediates resistance to numerous intracellular pathogens (such as *Leishmania*, *Mycobacterium*, and *Salmonella* [27]–[29] presumably due to its inability to transport iron from the phagosome to the cytosol [30]. Thus, RAW264.7 cells produce a *Salmonella* containing vacuole (SCV) the iron concentration of which might be high enough to inhibit *nrdHIEF* transcription via Fur.

Therefore, we repeated the same assays this time using a cell line transfected with this transporter (RAW264.7 *Nramp1^+/+^*) [31].

All mutants showed lower PI compared to those with the RAW264.7 cell line, but overall we obtained similar results. Neither the *nrdEF* (PI = 1.204), nor the *nrdDG* mutant (PI = 1.585) had significant differences when compared to the wild-type strain (PI = 1.239), and the *nrdAB* mutant (PI = 0.143) behaved very much alike in both cell lines (Figure 7A).

Nevertheless, when the RAW264.7 cell line was treated with 65 µM of the iron chelator 2,2′-dipyridyl (DIP), the *nrdAB* mutant containing an additional *nrdHIEF* copy showed a significantly higher PI than that of the untreated assay or the RAW264.7 *Nramp1^+/+^* cell line (Figure 7B).

The *nrdAB* mutant bearing a single *nrdHIEF* copy (IG137) was also analyzed in both RAW264.7 and RAW264.7 *Nramp1^+/+^* but at shorter times: 4, 6 and 24 hours post-infection (p.i.). As shown in Figure 7C, this mutant was able to maintain its viability up to 6 hours p.i., eventually dying (at 24 h p.i.) regardless of the cell line used.

**Figure 8:**
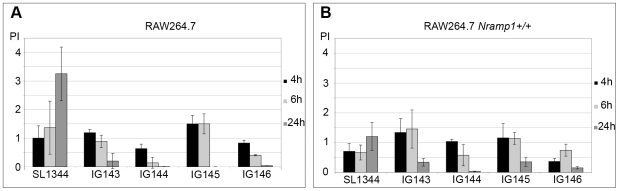
Role of *nrdHIEF* during macrophage infection. Proliferation indexes (PI) at 4, 6 and 24 h p.i. for wild-type, *ΔnrdABΔnrdR* (IG143) and *ΔnrdABΔfur* (IG144) double mutant strains and *ΔnrdABΔnrdDGΔnrdR* (IG145) and *ΔnrdABΔnrdDGΔfur* (IG146) triple mutant strains infecting (**A**) RAW264.7 and (**B**) RAW264.7 *Nramp1^+/+^*. Results are the mean values of at least three independent experiments. Error bars represent the standard deviation of the mean. Significant differences were determined by Student's *t* test for proliferation indexes with P<0.05.

Our next step was to assess whether the double (IG143 and IG144) and triple (IG145 and IG146) mutants experienced the same phenomenon. Figure 8 shows that during the early stages of infection these mutants were also able to maintain their viability growing at the expense of *nrdHIEF* overexpression, although there were no significant differences in their proliferation indexes and those of the *nrdAB* mutant. The effect of single *nrdR* or *fur* mutants on growth rates and macrophage infection was also evaluated as an additional control (Figures S3 and S4).

### Role of RNRs during infection of epithelial cells

In order to further investigate the involvement of each RNR class in the pathogenicity of *Salmonella* we analyzed invasion and proliferation inside epithelial cells.

Table 1 shows the invasion and proliferation indexes (%) of the different mutants inside a HeLa epithelial cell line.

**Table 1:** Invasion and proliferation indexes of wild-type and mutant strains infecting HeLa epithelial cell cultures.

Strain	Invasion Index HeLa*	Proliferation Index HeLa^‡^
SL1344	100±27.67	25.86±3.27
IG137 (Δ*nrdAB*)	0.027±0.019^#^	ND
IG138 (Δ*nrdEF*)	65.27±3.01	29.39±13.16
IG139 (Δ*nrdDG*)	130.94±92.45	23.51±1.90
IG140 (Δ*nrdEF*Δ*nrdDG*)	166.72±22.12	24.34±9.11
IG143 (Δ*nrdAB*Δ*nrdR*)	1.66±0.38^#^	0.30±0.30^#^
IG144 (Δ*nrdAB*Δ*fur*)	0.08±0.06^#^	0.027±0.04^#^
IG145 (Δ*nrdAB*Δ*nrdDG*Δ*nrdR*)	ND	ND
IG146 (Δ*nrdAB*Δ*nrdDG*Δ*fur*)	0.14±0.05^#^	0.62±0.35^#^

**Footnotes.** *Percentage of bacteria invading HeLa cells in 20 min and surviving the gentamicin protection assay. Viable counts were seeded at 2 h p.i. Values are normalized according to the wild-type strain (0.76±0.211). ^‡^Viable intracellular bacteria at 24 h p.i. versus viable intracellular bacteria at 2 h p.i. All results are the mean value of triplicates of three independent experiments. ^#^Significant values compared to the wild-type strain (p<0.05). ND: Not detected in any of the experiments.

Our results indicate that *nrdAB* was essential for invasion and proliferation of *Salmonella* inside HeLa epithelial cells, and given the assayed conditions neither *nrdHIEF* nor *nrdDG* seemed to have a relevant role in dNTP synthesis.

## Discussion

In this work we have studied the transcriptional regulation of the class Ia, class Ib and class III ribonucleotide reductases present in *S.* Typhimurium in response to Fur and NrdR transcriptional factors, and evaluated the role of each class during the course of an infection.

The presence of a poorly expressed class Ib enzyme in *S.* Typhimurium and *Enterobacteriaceae* in general has been a very puzzling issue since it was first described [32]. It has previously been shown that *nrdHIEF* may have a role during oxidative stress, although it does not seem to be regulated by either SoxRS or OxyR, the main regulators of the oxidative stress in enterobacteria [18]. *nrdHIEF* has also been suggested to respond to intracellular iron levels in a Fur-dependent fashion [8], [9].

In this work we have proven that Fur is indeed a transcriptional regulator of *nrdHIEF* in *S.* Typhimurium, normally repressing its expression and causing an upregulation under iron depleted growth (data not shown). We have also identified a Fur recognition sequence [33] (Figure 4A) located at position -309 within the *nrdHIEF* promoter region. Site-directed modification of the Fur box leads to a transcriptional upregulation of *nrdHIEF* similar to that caused by the Fur mutant. Furthermore, we have shown Fur binding to both this sequence and to its own promoter, indicating a direct role of the Fur protein in the regulation of class Ib *nrd* genes as well as in the autoregulation of *fur* expression similarly to what has previously been described in *E. coli* [23].

It has recently been suggested that a novel global regulator, termed NrdR, controls the overall expression of all *nrd* genes in a wide variety of microorganisms. NrdR was first described in *Streptomyces coelicolor* [12] as a regulator of the class Ia and class II RNRs present in this microorganism. Later on, Rodionov and Gefland reported the presence of putative NrdR boxes upstream from *nrd* genes in many different species of microorganisms [22]. It has been suggested that NrdR may regulate *nrd* expression in response to the intracellular pools of ATP/dATP, a hypothesis supported by the presence of an ATP-cone in this protein similar to that of the allosteric activity site of R1 [34].

Our results show that NrdR indeed regulates transcription of class Ia, class Ib and class III RNRs in *S.* Typhimurium LT2 and it has a major effect on *nrdHIEF* transcription. These results are in agreement with those found in *E. coli* [11].

We have also shown that the upregulated expression of both *nrdAB* and *nrdHIEF* upon addition of hydroxyurea is mediated by NrdR. Hydroxyurea is a growth-inhibitor capable of scavenging the tyrosyl radical found in the small subunit of class I RNRs, thus impairing its catalytic activity [16], [20], [35]. We believe that the scavenging activity of hydroxyurea alters the intracellular ATP/dATP pools, which are sensed by NrdR and cause its release from *nrd* promoters, leading to an increased expression of class I RNRs. Since HU cannot scavenge the glycyl radical of class III enzymes it should not alter wild-type *nrdDG* expression either in aerobic or anaerobic conditions, and we believe that the small increase observed in Figure 3 is mainly due to HU acting upon class I enzymes and activating NrdR release (note that FNR is also needed for *nrdDG* expression [10]). These results reinforce the role of NrdR as a global *nrd* regulator that mimics the allosteric responses of RNR at a transcriptional level.

Our results also show that *nrdHIEF* expression is independently regulated by Fur and NrdR, since an additive effect can be observed for the NrdR/Fur double mutant (Figure 5B).

In an attempt to characterize the direct binding of NrdR to *nrd* promoters, we specifically modified each of the putative NrdR recognition sequences described by Rodionov and Gefland [22]. Mutation of these sequences unveiled a complex regulatory mechanism since not all mutated boxes showed increased promoter expression.

In the *nrdAB* promoter, mutation of NrdRBox2 results in the same transcriptional levels as those of the NrdR mutant strain. The NrdRBox1 mutant and the double mutant, however, do not achieve the same levels detected with a *ΔnrdR* mutation, albeit they show increased transcriptional levels compared to the wild-type strain. The first nucleotides of the NrdR1 box overlap with the -10 sequence and even though we were careful not to modify them, it is possible that this mutation somehow affects RNA polymerase binding. Neither can we discard the presence of additional regulatory sequences within this region.

More disturbing is the observed lack of *nrdHIEF* expression for the NrdR2 box mutant. Again, modification of the putative recognition sequence might have altered the overlapping -10 box and prevented RNA polymerase from binding to the *nrdHIEF* promoter sequence. The increased expression of the double mutant, however, rules out this scenario and more likely the NrdR2 box is not involved in NrdR binding and promoter repression.

In the *nrdDG* promoter sequence, the NrdR1 box overlaps a few nucleotides from one of the FNR (fumarate nitrate reduction) recognition sites. FNR is a transcriptional regulator which activates transcription of some genes involved in anaerobic metabolism an represses the expression of some other genes needed during aerobic growth [36], [37]. If the NrdR1 box is mutated, NrdR cannot bind and therefore FNR is able to bind to its own recognition site, which could explain the higher expression levels found in NrdR1 box mutants (similar to those of the wild-type) compared to NrdR2 box mutants.

Electrophoretic mobility shifts assays could not be conducted for NrdR since the overproduced proteins were continuously recovered in the insoluble fraction upon purification, regardless of the elution conditions used (not shown).

There is no information available about the exact mechanism of NrdR repression. It is not well understood whether NrdR interaction with the promoter causes a direct blocking of RNA polymerase by steric hindrance or if binding of NrdR dimers to both boxes (note that NrdR boxes are palindromic sequences) causes DNA to bend impairing RNA polymerase binding.

Torrents *et al.* [11] suggested a more predominant role for the NrdR2 box of both *E. coli* and *S. Typhimurium* on the grounds of sequence conservation and the lack of mobility shifted bands when using a probe containing the *nrdA* promoter region with a mutated NrdR2 box. Our site-directed mutants corroborate these findings for the class Ia enzyme but NrdR regulation of the class Ib and class III genes requires further evaluation.

In the present study we also attempted to elucidate the actual role of the poorly transcribed class Ib reductase. Previous studies indicate overexpression of *nrdHIEF* under circumstances that might resemble those encountered during the course of an infection [8], [16], [18] and, therefore, we evaluated the contribution of each RNR enzyme in macrophage proliferation and cellular invasion.

Our results indicate that neither NrdEF nor NrdDG are essential for the growth of *Salmonella* inside macrophages and epithelial cell lines, being the class Ia enzyme the sole responsible for the replication of *Salmonella* in cultured cells, a situation that resembles what has been found *in vitro* [16].

An *nrdAB* mutant overexpressing *nrdHIEF*, however, is able to grow under standard laboratory conditions, indicating some ribonucleotide reductase activity and, when used to infect macrophage cell lines, it is able to maintain certain growth during the first hours of infection, eventually dying.

When growing in the lab, these mutants present evident growth deficiencies, tend to form filaments and need constant aeration, a phenomenon also described in an *E. coli* mutant with a *Mud1* insertion in *nrdB* growing in aerobiosis [38]. Similarly, an *nrdDG* mutant growing in strict anaerobiosis also forms filaments presumably due to a deficiency in DNA synthesis [39].

This growth deficiency may explain why all these *nrdAB* mutants are unable to invade epithelial cells since their fitness is severely reduced, but we believe *nrdHIEF* activity might have a role during the early stages of infection (4–6 hours post-infection).

Macrophages possess defense mechanisms against intracellular pathogens based on antimicrobial peptides, ribosomal enzymes, and radical generation by NADPH oxidase and inducible nitric oxide synthase (iNOS) [40]. NADPH oxidase is responsible for the respiratory burst, reducing oxygen to superoxide ion and this radical is the precursor of more reactive oxygen species (ROS) [41]. NADPH oxidase is the first enzyme to mediate the bactericide effect produced by the oxidative burst during the initial stages (2–4 h p.i.) [42], [43]. iNOS would participate in the nitrosative burst after NADPH oxidase to apply a sustained bacteriostatic effect [42], [44].

It has been described that these radicals damage membranes, enzymes and DNA. In fact, *S.* Typhimurium recombination deficient mutants (*recA* and *recBC*) are avirulent and sensitive to the oxidative burst of macrophages [43]. Hydrogen peroxide also participates in the oxidative burst and has been described as an inductor of *nrdHIEF* expression in *E. coli* [17], [18].

We suggest that *nrdHIEF* is needed to provide an extra supply of dNTPs to the cell when they are most needed: during cellular stress and DNA damage.

A similar dNTP extra supply has been described in the strict anaerobe *Bacteroides fragilis*, which has both class I and class III enzymes. Despite being an anaerobe, it can tolerate a certain amount of oxygen during extensive periods of time. This tolerance is due to a class Ia enzyme that synthesizes dNTPs enabling DNA repair and growth recovery after continuous exposure to oxygen [45].

Microarray profiling studies also indicate an OxyR-independent overexpression of *nrdHIEF* in *E. coli* in response to hydrogen peroxide [17] and, more recently, Hautefort *et al.* ([46]) showed induced *nrdHIEF* transcription during the early stages of macrophage infection but not in epithelial cell lines, thus corroborating our findings.

During oxidative stress, formed radicals can oxidize essential divalent cations, such as Fe^2+^, normally used as cofactors in many proteins. Hydrogen peroxide is able to oxidize the 4Fe-4S clusters present in many proteins to 3Fe-4S clusters. Iron is then free to act through Fenton reaction and cause DNA damage [47], [48]. In *E. coli* hydroxiperoxidase mutants treated with micromolecular levels of hydrogen peroxide, Fur is unable to control the free iron levels since oxidation of Fe^2+^ to Fe^3+^ by hydrogen peroxide inactivates its repressor activity. Thus, the oxidative burst and the subsequent DNA damage that takes place during the early infection most likely triggers *nrdHIEF* expression by means of both Fur and NrdR, helping to overcome the host defenses.

This is the first time that the role of different RNR classes during the course of an infection has been evaluated in *Enterobacteriaceae* and, although class Ia enzymes consolidate their essential function, a plausible role for class Ib RNR is suggested.

## Materials and Methods

### Bacterial strains, plasmids and growth conditions

Bacterial strains and plasmids used in this study are listed in Table S1. Cultures were typically grown in Luria-Bertani broth (LB; [49]) at 37°C. Cultures for preparation of electrocompetent cells were grown in 2xYT medium at 37°C. Solid media were prepared using 1.5% agar. Antibiotics were used at the following concentrations: kanamycin, 50 µg ml^−1^; ampicillin, 50 µg ml^−1^; spectinomycin, 200 µg ml^−1^; rifampicin, 75 µg ml^−1^; chloramphenicol, 35 µg ml^−1^.

Anaerobic growth was achieved using LB + sodium sulphate (3.2 mM) or LB + nitrates (KNO_3_ 0.04 M + sodium molybdate 10^−3^ mM). Tubes were filled to the top without leaving any air bubbles. Strains were grown on solid media in LB + nitrates inside Anaerocult^®^ (Merck) bags with an anaerobic indicator (Anaerotest^®^, Merck).

### General genetic techniques

General DNA manipulations were done by standard procedures [50]. Transductions in *S. enterica* serovar Typhimurium were carried out using a high-transduction derivative of phage P22*int*-7 as described by Miller [49]. Conjugations were carried out as described by de Lorenzo *et al.* [51].

### Construction of reporter fusions

Reporter fusions were constructed as described by de Lorenzo *et al.* [51]. Briefly, DNA fragments of approximately 500 bp containing the 5′ end of the *nrdAB*, *nrdEF* or *nrdDG* genes and the upstream regulatory regions were ligated into the *EcoRI/BamHI* sites of plasmid pUJ8, yielding transcriptional fusions between these fragments and the *lacZ* gene. All constructs were confirmed by PCR and DNA sequencing. Fusions were then transferred to plasmids pUT-miniTn*5-*Km2 or pUT-miniTn*5-*Sm/Spc by *Not*I digestion and introduced into the *E. coli* conjugative strain S17-1 λ*pir*. Biparental conjugation between S17-1λ*pir* and recipient strains was used to obtain transposition and insertion of the fusions within the *Salmonella* chromosome, generating the strains listed in Table S1.

### Beta-galactosidase activity assays

β-Galactosidase activities expressed from *nrdDG-lacZ* fusions were assayed according to the method of Miller [49] using cultures grown in LB broth either oxically or anoxically (in screw-cap tubes filled to the neck with 3.2 mM sodium sulfide). The quoted specific activities (Miller Units) are averages of triplicate samples of at least three independent cultures.

### Construction of *nrdR* and *fur* null mutants

*nrdR* and *fur* null mutants were constructed using the method described by Wanner and Datsenko [52]. The coding region of *nrdR* and *fur* was replaced by a kanamycin resistance cassette from pKD4. Mutated genes were subsequently transduced to *Salmonella enterica* serovar Typhimurium LT2 strains bearing the *nrdAB*::*lacZ*, *nrdEF*::*lacZ* and *nrdDG*::*lacZ* transcriptional fusions (see Table S1). The unmarked mutants were also obtained as described by Wanner and Datsenko.

### Complementation assays

*nrdR* and *fur* were amplified by PCR using primer pairs containing a ribosomal binding site sequence from *E. coli* and the *SacI* (forward primers) and *XbaI* (reverse primers) restriction sites. The resulting fragments were purified and digested with *SacI* and *XbaI* and inserted into pBAD_33_Cm [53] to generate pIG85 (*nrdR*) and pIG89 (*fur*), which were subsequently electroporated into *S.* Typhimurium LT2 Δ*nrdR* or *Δfur* containing the desired *nrd-lacZ* reporter fusions. Plasmid expression was induced with 0.2% L-arabinose after 30 minutes.

### Site-directed mutagenesis of NrdR recognition sequences

NrdR1 and NrdR2 boxes in the promoter regions of *nrdAB*, *nrdHIEF* and *nrdDG* were mutagenized by overlap extension PCR [54]. Briefly, a first PCR was performed to amplify the 5′ and 3′ ends of the promoter regions using primers that introduced the desired mutations. A second PCR was then performed using the 5′ and 3′ amplicons as a template and external primers to amplify the full length product. The resulting amplicons were cloned into the pGEM-t easy vector (Promega) and used to construct *lacZ* reporter fusions as described above.

### Protein expression and purification of Fur

The *fur* gene was amplified by PCR from a *Salmonella enterica* serovar Typhimurium LT2 colony using a set of primers that introduced *NdeI* and *BamHI* restriction sites at the 5′ and 3′ ends of the amplicon, respectively. Following purification, it was inserted into the cognate sites of pET22a (Novagen) to introduce a C-terminal 6xHis tag. The final construct was then transformed into *E. coli* BL21(DE3) and grown overnight at 37°C in LB medium containing 50 µg ml^−1^ carbenicillin. Overnight cultures of *E. coli* BL21(DE3) bearing the pET22b-*fur* plasmid were diluted 1/100 in LB with 50 µg ml^−1^ carbenicillin and grown at 37°C with shaking until they reached an A_600_∼0.5. The culture was then inoculated with isopropyl-β-D-thiogalactopyranoside (IPTG) (Sigma) to a final concentration of 0.5 mM and grown for 4 hours at 37°C. Cells were harvested by centrifugation at 3,000xg for 10 min at 4°C and the cell pellet stored at −70°C.

The pellet was resuspended in buffer A solution (20 mM PBS, 0.5 M NaCl and 20 mM imidazole) and extensively sonicated. The cell lysate was centrifuged at 38,000xg for 1 hour at 4°C and the supernatant was loaded into a HisTrap HP Ni^2+^-affinity column (Amersham biosciences). The Fur protein was eluted at 330 mM imidazole and dialyzed against 50 mM Tris-HCl pH 7.5, 300 mM NaCl and 15% glycerol buffer. Dialyzed protein was further concentrated in Centriplus YM-10 (Millipore) and stored at −80°C. The quality and the concentration of the samples were assessed by SDS-PAGE and the Bradford method, respectively [55].

### Electrophoretic mobility shift assay

A DNA probe of 478 bp containing the promoter region of *nrdHIEF* was amplified by PCR and cloned into the pGEM-t easy vector. The same was done with a DNA probe of 474 bp containing the *fur* promoter region. The DNA probes were 3′dig-ddUTP labelled using a terminal transferase kit (Roche). Binding reactions were carried out in a final volume of 20 µl containing binding buffer (10 mM Tris-HCl pH 8, 5% glycerol, 100 µM MnCl_2_, 1 mM MgCl_2_, 40 mM KCl, 0.1 mg/ml BSA), 3′dig-ddUTP-labelled DNA (20 pg), and purified Fur protein (ranging from 0 to 1.5 µg of protein). Binding reactions were incubated 20 min at 37°C and loaded into a 5% polyacrylamide gel (30∶0.8 acrylamide:bisacrylamide w/v) in 0.5× TBE buffer. The gel was electro-blotted onto a positively charged nylon membrane (Roche), UV-cross-linked and developed by chemiluminescence and colorimetric procedures according to the manufacturer's protocols (Roche Diagnostics).

### Construction of *nrd* mutants

*nrdHIEF* and *nrdDG* unmarked mutants were constructed according to Wanner and Datsenko [52] originating the *S.* Typhimurium strains IG138 and IG139 (see Table S1). An entire fragment of 3103 bp containing both the *nrdE* and *nrdF* coding regions was deleted in strain IG138, and in IG139 both the *nrdD* and *nrdG* coding regions are missing.

The *nrdAB* mutant strain had been previously constructed in our lab. It is an LT2 strain containing an *nrdA'::ΩSpc'nrdB* mutation and an extra *nrdEF* copy inserted elsewhere in the chromosome (merodiploid) allowing aerobic growth. *nrdA'::ΩSpc'nrdB* from this strain was transduced to the intermediate strain (SL1344 *ΔnrdEF::Km*) obtained during construction of *ΔnrdEF* with the Wanner and Datsenko method. A second *nrdAB* mutant strain was also obtained by transducing *nrdA'::ΩCm'nrdB* from strain IG1 pIG8 into SL1344 wild-type in anaerobic conditions. This mutant contains a deletion in the *nrdAB* operon leaving only 560 bp of the 5′ end (168 bp corresponding to *nrdA* and 392 bp corresponding to the non-coding region in the 5′ end) as well as 440 bp of the 3′ end of *nrdB*. Termed IG137, this strain cannot grow under aerobic conditions.

A *ΔnrdEF/ΔnrdDG* double mutant strain (IG140) was obtained by transducing *ΔnrdDG::km* to strain IG138 and then eliminating the antibiotic marker.

Since the double *ΔnrdAB/ΔnrdDG* mutant cannot grow either in the presence or absence of oxygen, it was necessary to force *nrdHIEF* overexpression to allow its growth in aerobiosis. First we constructed the *ΔnrdAB/ΔnrdR* (IG143) *and ΔnrdAB/Δfur* (IG144) double mutants in anaerobiosis and afterwards we transduced the *ΔnrdDG::km* mutation to originate strains IG145 and IG146, respectively.

### Replication of *S.* Typhimurium inside RAW264.7 macrophages (Gentamicin protection assays)

RAW264.7 (ATCC #TIB-71) and RAW264.7 *Nramp1^+/+^* [31] macrophages were harvested and seeded at 5×10^5^ cells per well in 24-well tissue culture plates, allowed to adhere and grown to 80% confluence in DMEM (with Glutamax^®^) supplemented with FBS 10% for 24 hours. Bacterial mutant strains were grown overnight at 37°C without agitation in order to achieve stationary phase. Overnight cultures were diluted to obtain a MOI of 10 and incubated with the cell lines for 20 minutes at 37°C and 10% CO_2_. Cells were washed twice with PBS to eliminate extracellular bacteria. 500 µl of DMEM with 100 µg ml^−1^ gentamicin were added to further incubate the cells during 1 h 30 min at 37°C. Cells were then washed twice with PBS and a fraction of the plate was treated with Triton X-100 1% to obtain intracellular bacteria. DMEM containing 10 µg ml^−1^ gentamicin was added to the remaining wells and the plate was incubated for 4, 6, or 24 hours. Cells treated with Triton X-100 1% were incubated for 10 min at 37°C and resuspended with DMEM and used to perform viable counts of intracellular bacteria. After 8 and 24 h post-infection cells were also treated with Triton X-100 to obtain intracellular viable cells. Proliferation indexes (PI) were calculated as CFU/ml at the various time points post-infection (p.i) divided by CFU/ml at 2 h p.i. Significant differences were determined by Student's *t* test for proliferation indexes with P<0.05.

### Invasion assay of epithelial cell lines

This procedure is similar to the gentamicin protection assay but with subtle differences. HeLa cells (ATCC #CCL-2) were seeded at 4–5×10^4^ cells per well in 24-well tissue culture plates, allowed to adhere and grown to 80% confluence in MEM supplemented with FBS 10% and glutamine for 24 hours. The MOI used in these assays was 50–100. Cells were infected for 20 min and the procedure was the same as in macrophage assays with the only difference that cells were harvested at 2 h and 24 h p.i.

Invasion indexes were calculated as CFU/ml at 2 h p.i divided by CFU/ml of the input. Proliferation indexes were calculated as before. Significant differences were determined by Student's *t* test for invasion indexes with P<0.05.

## Supporting Information

Table S1 Bacterial strains and plasmids used in this study.
(0.12 MB DOC)

Figure S1 Effect of *Δfur* mutation on *nrdAB* expression β-galactosidase activity is expressed in Miller Units (MU) for the wild-type strain (Wt) and mutant *Δfur* strain (Fur^-^).
(0.05 MB TIF)

Figure S2 Effect of *nrd* mutants on growth rate. (A) Growth curve of the *nrdA′::ΩCm′nrdB Δfur (ΔAB-Δfur), nrdA′::ΩCm′nrdB ΔnrdDG Δfur (ΔAB-ΔDG-Δfur), nrdA′::ΩCm′nrdB ΔnrdDG ΔnrdR (ΔAB-ΔDG-ΔR)* and *Wt* (SL1344) strains from *S. Typhimurium* growing under aerobic conditions together with (B) viable counts.
(0.10 MB TIF)

Figure S3 Effect of NrdR and Fur mutants on growth rate. Growth curves for the Wt, NrdR and Fur mutant *Salmonella* strains.
(0.06 MB TIF)

Figure S4 Effect of NrdR and Fur on macrophage infection. Proliferation indexes in RAW264.7 macrophage cultures for the Wt, NrdR and Fur mutant *Salmonella* strains.
(0.04 MB TIF)
