# Supplementary material for: Ribonucleotide Reductases of Salmonella Typhimurium: Transcriptional Regulation and Differential Role in Pathogenesis
Source: PLoS One. 2010 Jun 25;5(6):e11328. doi: 10.1371/journal.pone.0011328 (PMC2892513; doi:10.1371/journal.pone.0011328)
Supplement: Table S1 — Bacterial strains and plasmids used in this study. (0.12 MB DOC) [file pone.0011328.s001.doc]

Supporting information

**TABLE S1.** Bacterial strains and plasmids used in this study.

| Strain / Plasmid | Genotype / sequence | | Source |
| --- | --- | --- | --- |
| ***E. coli* strains** |  | |  |
| BW25113 | Δ(*araD*-*araB*)*567* Δ*lacZ4787* (::rrnB-3) *lacIp*- *4000* (*lacI^Q^*) λ^-^ *rph-1* Δ(*rhaD-rhaB*)*568* *hsdR514* | | [52] |
| BW25141 | Δ(*araD-araB*)*567* Δ*lacZ4787* (::rrnB-3) *lacIp-* *4000* (*lacI^Q^*) Δ(*phoB-phoR*)*580* λ^-^ *galU95* Δ*uidA3::pir^+^* *recA1* *endA9*(del-ins)*::FRT* *rph-1* Δ(*rhaD-rhaB*)*568* *rrnB-3* *hsdR514* | | [52] |
| BT340 | Δ(*argF-lac*)*169* *φ80dlacZ58*(M15) *glnV44*(AS) λ^-^ *rfbD1* *gyrA96*(NalR) *recA1* *endA1* *spoT1* *thi-1* *hsdR17* pCP20 | | [52] |
| DH5α | *endA1 hsdR17*(*r^-^m^-^*) *supE44 thi-1* *recA1 gyrA96 relA1* Δ(*lacZYA-argF*)*U169 deoR* [*φ80 dlac* Δ (*lacZ*)*M15*] | | Clontech |
| MC1061 λ*pir* | Δ*lacX74 hsdR mcrB araD139* Δ(*araABC-leu*)*7679 galU galK rpsL thi* | | Laboratory stock |
| S17-1 λ*pir* | S17-1 lysogenized with λ *pir* bacteriophage | | [51] |
| ***S. enterica* serovar Typhimurium strains** | | | |
| SL1344 |  | | Laboratory stock |
| LT2 |  | | ATCC#700720 |
| LT2 Rif^R^ |  | | Laboratory stock |
| SL1344 Rif^R^ |  | | This work |
| IG102 | LT2 *nrdR* gene disrupted with the Km cassette from pKD4 | | This work |
| IG103 | LT2 unmarked *nrdR* mutant | | This work |
| LT2 *nrdAB::lacZ* | Mini-Tn*5* Sm/Spc-*nrdAB*::*lacZ* (Spc^R^) | | Laboratory stock |
| LT2 EA445.1 | Mini-Tn*5* Sm/Spc-*nrdEF*::*lacZ* (Spc^R^) | | Laboratory stock |
| LT2 *nrdDG::lacZ* | Mini-Tn*5*Km-*nrdDG*::*lacZ* (Km^R^) | | Laboratory stock |
| IG104 | *ΔnrdR* transduced to LT2 *nrdAB*::lacZ (Spc^R^) | | This work |
| IG105 | *ΔnrdR* transduced to LT2 EA445.1 | | This work |
| IG106 | *ΔnrdR* transduced to LT2 *nrdDG*::lacZ (Km^R^) | | This work |
| IG107 | IG104 with pBAD_33_*nrdR* | | This work |
| IG108 | IG105 with pBAD_33_*nrdR* | | This work |
| IG109 | IG106 with pBAD_33_*nrdR* | | This work |
| IG110 | LT2 *RBoxAB1-lacZminiTn5-Km2* | | This work |
| IG111 | LT2 *RBoxAB2-lacZminiTn5-Km2* | | This work |
| IG112 | LT2 *RBoxAB1AB2-lacZminiTn5-Km2* | | This work |
| IG113 | LT2 *RBoxEF1-lacZminiTn5-Km2* | | This work |
| IG114 | LT2 *RBoxEF2-lacZminiTn5-Km2* | | This work |
| IG115 | LT2 *RBoxEF1EF2-lacZminiTn5-Km2* | | This work |
| IG116 | LT2 *RBoxDG1-lacZminiTn5-Km2* | | This work |
| IG117 | LT2 *RBoxDG2-lacZminiTn5-Km2* | | This work |
| IG118 | LT2 *RBoxDG1DG2-lacZminiTn5-Km2* | | This work |
| IG128 | LT2 *fur* gene disrupted with the Km cassette from pKD4 | | This work |
| IG129 | LT2 *fur* gene disrupted with the Cm cassette from pKD3 | | This work |
| IG130 | LT2 unmarked *fur* mutant | | This work |
| IG131 | *Δfur* transduced to LT2 EA445.1 | | This work |
| IG132 | IG131 with pBAD_33_Fur | | This work |
| IG133 | LT2 *MutFurBoxEF-lacZminiTn5-Km2* | | This work |
| IG134 | LT2 Δfur ΔnrdR | | This work |
| IG135 | *nrdEF::lacZ* transduced to IG134 | | This work |
| IG147 | LT2 *nrdA’::ΩSpc’nrdB* *nrdEF::Km* merodiploid | | Laboratory stock |
| IG1 pIG8 | LT2 *nrdA’::ΩCm’nrdB* pIG8 | | Laboratory stock |
| IG136 | SL1344 *nrdA’::ΩSpc’nrdB* *nrdEF::Km* merodiploid | | This work |
| IG137 | SL1344 *nrdA’::ΩCm’nrdB* (anaerobic growth) | | This work |
| IG138 | SL1344 *ΔnrdEF* | | This work |
| IG139 | SL1344 *ΔnrdDG* | | This work |
| IG140 | SL1344 *ΔnrdEF ΔnrdDG* | | This work |
| IG141 | SL1344 *ΔnrdR* | | This work |
| IG142 | SL1344 *Δfur* | | This work |
| IG143 | SL1344 *nrdA’::ΩSpc’nrdB ΔnrdR* | | This work |
| IG144 | SL1344 *nrdA’::ΩSpc’nrdB Δfur* | | This work |
| IG145 | SL1344 *nrdA’::ΩSpc’nrdB ΔnrdDG ΔnrdR* | | This work |
| IG146 | SL1344 *nrdA’::ΩSpc’nrdB ΔnrdDG Δfur* | | This work |
| **Plasmids** |  | |  |
| pKD3 | | *oriR6Kγ bla*(Ap^R^) *cat rgnB*(Ter) | [52] |
| pKD4 | | *oriR6Kγ bla*(Ap^R^) *kan rgnB*(Ter) | [52] |
| pCP20 | | *ts-rep* [*FLP*] [*cI857*](lambda)(ts) *bla*(Ap^R^) *cat* | [52] |
| pKD46 | | *araBp-gam-bet-exo* *bla*(Ap^R^) *repA101*(ts) *oriR101* | [52] |
| pGEM®-t easy | | Cloning vector | Promega |
| pBAD_33_Cm | | Ara P_BAD_ promoter high expression vector Cm^R^ | [53] |
| pIG8 | | pBAD_33_*nrdHIEF* | Laboratory stock |
| pIG85 | | pBAD_33_+ *nrdR* ORF cloned in *SacI-XbaI* sites | This work |
| pIG89 | | pBAD_33_+ *fur* ORF cloned in *SacI-XbaI* sites | This work |
| pUJ8 | | Promoterless vector for making *lacZ* fusions Ap^R^ | [51] |
| pUT miniTn5 Km2 | | Mini-Tn*5* Km2 in plasmid pUT Ap^R^ Km^R^ | [51] |
| pUT miniTn5 Spc | | Mini-Tn*5* Spc in plasmid pUT Ap^R^ Spc^R^ | [51] |
| pET22b(+) | | Expression vector | Novagen |
