# Supplementary figures and images for: Ribonucleotide Reductases of Salmonella Typhimurium: Transcriptional Regulation and Differential Role in Pathogenesis
Source: PLoS One. 2010 Jun 25;5(6):e11328. doi: 10.1371/journal.pone.0011328 (PMC2892513; doi:10.1371/journal.pone.0011328)

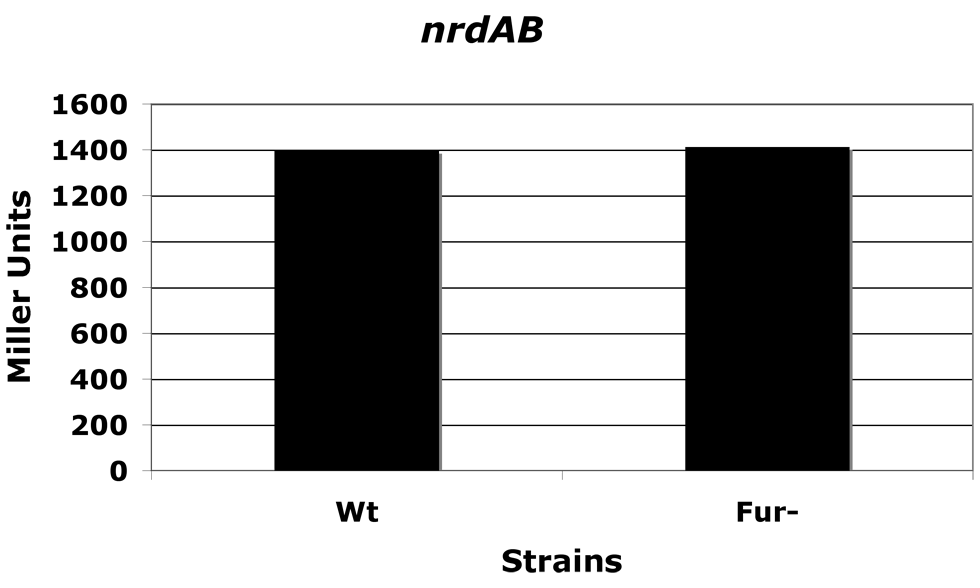

Supplement: Figure S1 — Effect of Δfur mutation on nrdAB expression β-galactosidase activity is expressed in Miller Units (MU) for the wild-type strain (Wt) and mutant Δfur strain (Fur-). (0.05 MB TIF) [file pone.0011328.s002.tif]

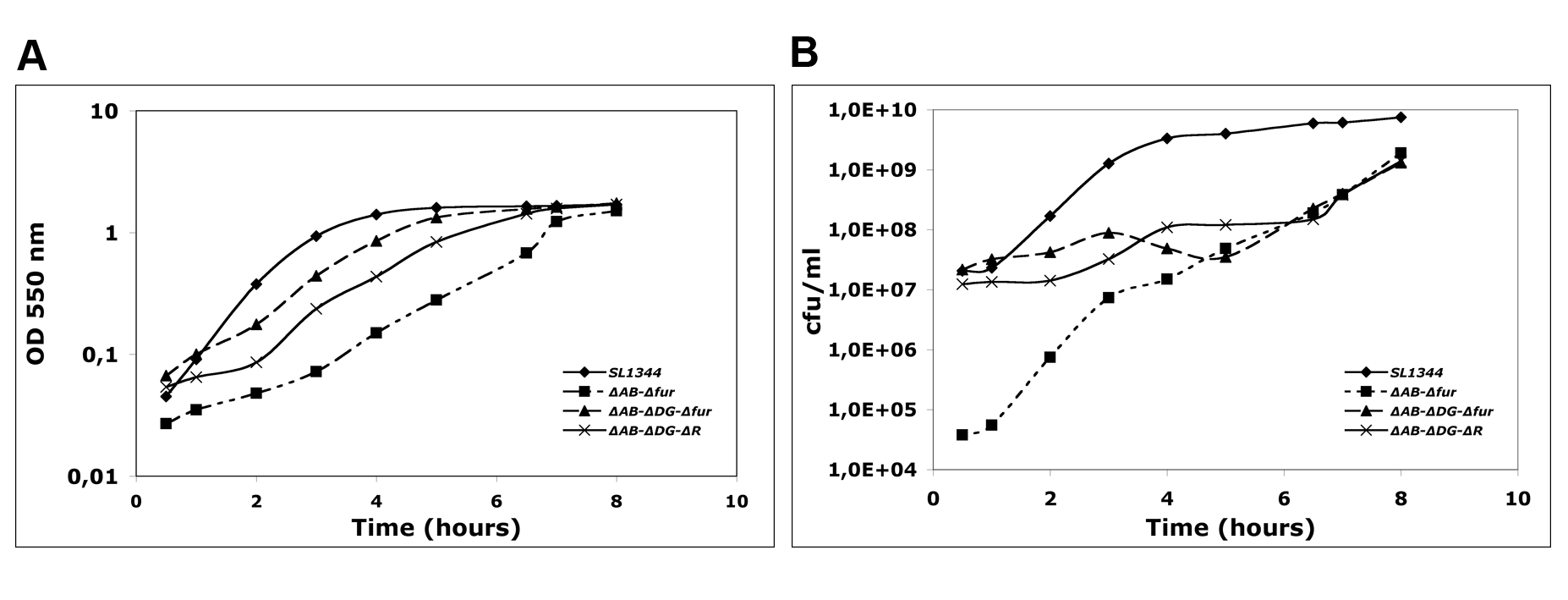

Supplement: Figure S2 — Effect of nrd mutants on growth rate. (A) Growth curve of the nrdA′::ΩCm′nrdB Δfur (ΔAB-Δfur), nrdA′::ΩCm′nrdB ΔnrdDG Δfur (ΔAB-ΔDG-Δfur), nrdA′::ΩCm′nrdB ΔnrdDG ΔnrdR (ΔAB-ΔDG-ΔR) and Wt (SL1344) strains from S. Typhimurium growing under aerobic conditions together with (B) viable counts. (0.10 MB TIF) [file pone.0011328.s003.tif]

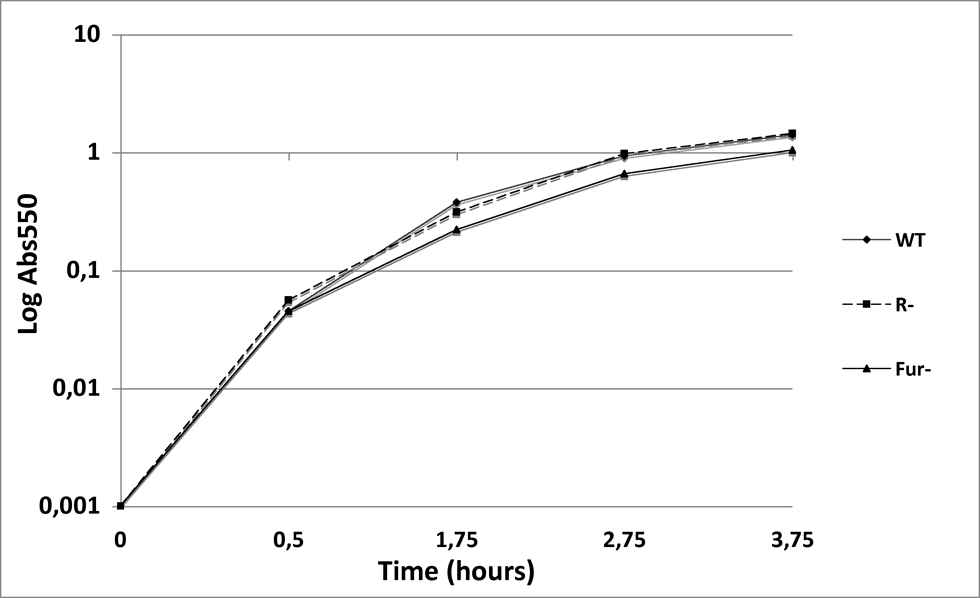

Supplement: Figure S3 — Effect of NrdR and Fur mutants on growth rate. Growth curves for the Wt, NrdR and Fur mutant Salmonella strains. (0.06 MB TIF) [file pone.0011328.s004.tif]

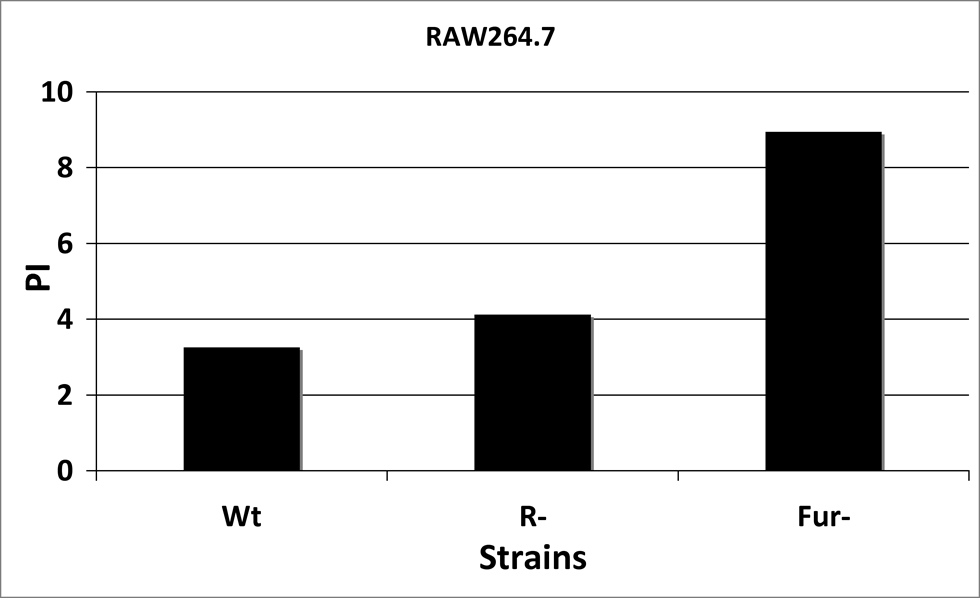

Supplement: Figure S4 — Effect of NrdR and Fur on macrophage infection. Proliferation indexes in RAW264.7 macrophage cultures for the Wt, NrdR and Fur mutant Salmonella strains. (0.04 MB TIF) [file pone.0011328.s005.tif]
